# The Effect of Myo-Inositol Supplementation During Pregnancy on Fetal and Maternal Outcomes: Results of the Myo-Inositol for the Prevention of Gestational Diabetes Mellitus (MiGDM) Randomized Double-Blind, Placebo-Controlled Pilot Trial

**DOI:** 10.7759/cureus.102259

**Published:** 2026-01-25

**Authors:** Gbemisola Okunoye, Chinnu George Samuel, Hala Abdullahi, Annalisa Terranegra, Ibrahim M Ibrahim

**Affiliations:** 1 Obstetrics and Gynaecology, Sidra Medicine, Doha, QAT; 2 Research, Sidra Medicine, Doha, QAT; 3 Endocrinology, Sidra Medicine, Doha, QAT

**Keywords:** gestational diabetes mellitus, insulin resistance, myo-inositol, neonatal outcomes, pregnancy outcomes, randomized controlled trial

## Abstract

Gestational diabetes mellitus (GDM) is a common pregnancy complication with significant maternal and neonatal risks, and its prevalence is particularly high in Qatar. Myo-inositol has been proposed as an insulin-sensitizing supplement that may reduce the incidence of GDM, but evidence is limited in this population. This study aims to evaluate the effect of antenatal dietary myo-inositol supplementation on the incidence of GDM among pregnant women in Qatar and to assess the impact on fetal and neonatal outcomes. Sidra Medicine, Qatar, conducted this prospective, randomized, double-blind, placebo-controlled pilot trial (2022-2023). The trial was registered with International Standard Randomised Controlled Trial Number (ISRCTN; protocol number ISRCTN16448440) and approved by the Institutional Review Board of Sidra Medicine (IRB Number: 1538656). Only 67 pregnant women were recruited, and 43 of them finished the trial till birth, despite the study's initial power to enrol 640 pregnant women (320 each group). This was due to funding-related early discontinuation. Eligible women were ≤16 weeks of gestation, planned to deliver at the study center, and had no pre-gestational diabetes. Participants were randomized to receive either myo-inositol supplementation (2 g twice daily) or a placebo until delivery. Data collection included maternal demographics, anthropometric measurements, and pregnancy and neonatal outcomes. The primary outcome was the incidence of GDM diagnosed by oral glucose tolerance test in both groups. Secondary outcomes included pregnancy and neonatal outcomes, along with predictors of insulin resistance (Homeostasis Model Assessment of β-cell function (HOMA B; %) and Homeostatic Model Assessment of Insulin Resistance (HOMA IR; index)). Although the study concluded before reaching target recruitment due to funding challenges, an analysis of the cumulative data was performed. Sixty-seven pregnant women were recruited, and 43 patients completed the study until delivery. There were 18 patients in the myo-inositol group and 25 in the placebo group. No significant difference was observed between the two groups in the incidence of GDM. Additionally, there were no significant differences between the two groups in the measures of insulin resistance (HOMA B or HOMA IR) and maternal and neonatal outcomes. Dividing the study population into those who developed GDM (16 patients) versus those who did not develop GDM (27 patients) revealed significantly higher fasting insulin levels (12.65 ± 7.29 µIU/mL vs 7.37 ± 3.24 µIU/mL, p=0.027) and increased insulin resistance measured by HOMA IR (3.05 ± 1.82 vs 1.54 ± 0.69, p=0.010) in the GDM group compared to the non-GDM group. However, there was no significant difference in HOMA B between the two groups. The GDM group had a lower gestational age at delivery (37.8 ± 3.89 weeks vs 39.21 ± 0.57 weeks, p=0.03) than the non-GDM group. There was no statistically significant difference in the incidence of GDM or the outcomes for mothers and newborns between the myo-inositol and placebo groups in this severely underpowered sample. This study was unable to definitively evaluate the original hypothesis due to the significant recruitment deficit and consequent lack of statistical power. Therefore, the results should be regarded as inconclusive, and we are unable to draw clear conclusions about the effectiveness of myo-inositol supplementation.

## Introduction

GDM is defined as any degree of glucose intolerance with onset or first recognition during pregnancy. It poses both maternal and fetal risks and is also associated with an increased long-term likelihood of developing type 2 Diabetes (T2DM). GDM is a growing public health issue, with its prevalence rising due to increasing obesity, insulin resistance, and advanced maternal age. It is the most frequent medical complication of pregnancy, and approximately 36% of pregnant women in Qatar experience GDM, making it a significant health concern. Evidence-based therapies and measures have been introduced in the past to improve outcomes for women with GDM during and beyond pregnancy, with some evidence that metformin treatment combined with lifestyle changes can delay or prevent diabetes in women with a history of GDM [1]. Despite significant progress, challenges remain with current therapies, including resistance to insulin injections or taking metformin pills, and non-compliance with dietary recommendations.

Myo-inositol, an isomer of inositol, is a naturally occurring sugar commonly found in cereals, corn, legumes, and meat. It is an insulin-sensitizing mediator that reduces plasma glucose levels and improves insulin sensitivity in polycystic ovary syndrome (PCOS), and it has been proposed as a dietary supplement with the potential to reduce the incidence of GDM in at-risk women [2-5]. In women with PCOS and metabolic dysfunction, myo-inositol has been demonstrated to increase insulin sensitivity by acting as a precursor of inositol phosphoglycans implicated in insulin signal transduction [4]. A study found that myo-inositol supplementation was associated with a reduction in the incidence of GDM and macrosomic babies in pregnant women with a parent with T2DM [6]. Investigators have confirmed the ubiquitous nature of myo-inositol as being present in the membranes of all living cells, and its role as one of the intracellular mediators of the insulin-signaling pathway associated with insulin sensitivity in T2DM [7].

The association of myo-inositol with lower plasma glucose levels and improved insulin sensitivity and ovulatory function in young women with PCOS makes it an ideal candidate for focused research in relation to the incidence of GDM [8]. A Cochrane study found that myo-inositol was associated with a lower incidence of GDM (relative risk (RR) 0.43, 95% confidence interval (CI) 0.29 to 0.64), though the impact on neonatal outcome was less clear [9]. It is uncertain whether this finding is generalizable in mixed and diverse populations, particularly those with an existing high incidence of GDM, such as Qatar. Findings from other studies evaluating the impact of myo-inositol supplementation during pregnancy on maternal and neonatal outcomes have shown mixed results [10]. Pregnancy-related hypertensive problems, cesarean birth, macrosomia, neonatal hypoglycemia, and long-term cardiometabolic illness in both the mother and the child are all linked to GDM [11].

It is possible that preventive measures like myo-inositol may show a lower relative effect when compared to groups at lower baseline risk because of Qatar's remarkably high baseline prevalence of GDM (around 36%). In some situations, obesity, genetic vulnerability, or more established insulin resistance may be the cause of dysglycemia, necessitating earlier commencement, larger dosage, or risk-stratified targeting in order to obtain quantifiable effect. Consequently, even while previous research has shown that myo-inositol supplementation lowers the incidence of GDM, it is still unclear if these benefits apply to groups with a significantly higher baseline risk of GDM.

Aim of the study

To determine whether antenatal myo-inositol supplementation, compared with placebo, reduces the risk of GDM in a prospective, randomized, double-blind, placebo-controlled pilot trial. We hypothesized that myo-inositol lowers the incidence of GDM (primary outcome, diagnosed by standard oral glucose tolerance test (OGTT) criteria). Secondary outcomes included maternal outcomes (gestational weight gain, need for metformin or insulin therapy, mode of delivery, and hypertensive disorders of pregnancy) and fetal/neonatal outcomes (large-for-gestational-age >95th centile, small-for-gestational-age <10th centile, macrosomia ≥4000 g, shoulder dystocia/birth injury, polyhydramnios, neonatal ICU (NICU) admission >24 h, neonatal hypoglycemia requiring intravenous dextrose, preterm birth <37 weeks of gestation, transient tachypnea of the newborn, and respiratory distress syndrome). We hypothesized that myo-inositol supplementation would lower the incidence of GDM. However, since the study population had a high baseline risk, we expected that any preventive effect would be modest and possibly insufficient to show a statistically significant reduction without earlier initiation, higher dosage, or targeted use in specific high-risk subgroups.

## Materials and methods

Study design

We carried out a prospective, randomized, double-blind, placebo-controlled pilot trial at Sidra Medicine Hospital in Qatar (2022-2023). Enrolled participants were divided equally between the intervention group (myo-inositol) and the control group (placebo). Participants were recruited based on the trial's inclusion and exclusion criteria. The trial was registered with International Standard Randomised Controlled Trial Number (ISRCTN; protocol number ISRCTN16448440) and approved by the Institutional Review Board of Sidra Medicine (IRB Number: 1538656).

Eligible participants were pregnant women presenting for antenatal booking at the trial center with ≤16 weeks of gestation, who had capacity to provide informed consent, no pre-gestational diabetes, a booking fasting plasma glucose <5.1 mmol/L (92 mg/dL), who were not using systemic steroids or metformin for any indication, had no history of bariatric surgery, had no active cancer (remission permitted), were not taking myo-inositol supplements, were not enrolled in another interventional trial, and had no polyhydramnios (unlikely before 16 weeks). The exclusion criteria were pre-gestational diabetes; fasting glucose ≥5.1 mmol/L; steroid use during pregnancy; metformin use for any condition (including PCOS); current myo-inositol supplementation; cancer not in remission; inability to consent; prior bariatric surgery; participation in another interventional trial; or presence of polyhydramnios. 

After randomization, baseline characteristics were compared between the groups. The prevalence of obesity (BMI >30 kg/m^2^), a recognized main risk factor for GDM, showed a statistically significant imbalance. The primary and secondary analyses did not do a multivariable adjustment for obesity or BMI due to the small sample size and potential for model instability.

Study procedure

*Randomization* 

Pregnant women were approached at their antenatal booking visit, and comprehensive information about the study was provided in English and Arabic, and written informed consent was obtained from participants who met the inclusion criteria. Study participants were randomized into intervention and control groups. The randomization was carried out with computer-generated numbers, and the blinding was ensured through identical packaging of the placebo and myo-inositol, at source by the pharmaceutical business Nutrilinea S.r.l (Gallarate (Varese), Italy), which handled the production and packaging of both the myo-inositol and placebo and was not involved in the study [12].

Study participants received either a placebo (2g twice daily) or myo-inositol (2g twice daily) with supplements that were dispensed monthly, and compliance was monitored through scheduled contacts, which were linked to regular antenatal hospital visits throughout pregnancy. The remaining study sachets were collected and logged on admission for delivery.

Monitoring and Follow-Up

The study participants continued their routine antenatal care in accordance with the Qatar national guidelines until delivery. An OGTT was performed between 24 and 28 weeks of gestation, linked to a scheduled clinic appointment. At the time of OGTT, fasting insulin and C-peptide levels were also measured.

Data on the course of pregnancy and pregnancy outcomes were collected along with lifestyle and dietary data using 24-hour recall and questionnaires. The study participants who withdrew from the study had their routine clinical data collected unless they explicitly withdrew consent.

Data Collection

Data were collected at baseline and at regular intervals using a combination of electronic health records or EHRs (CERNER™, Oracle Health, Missouri, US) and paper forms. Data collected included participant identifiers (study ID, date of birth, nationality, and ethnicity); pregnancy characteristics (gestational age, parity, prior pregnancy history, multiple gestations, congenital fetal abnormalities, and lifestyle exposures such as nutrition, exercise, and smoking); and anthropometry (weight and height). Randomization numbers were maintained in a restricted-access list available only to a designated study team member and the Principal Investigator. Case report forms (CRFs) were completed from source data in the Cerner EHR; any paper CRFs were subsequently transcribed into the REDCap database (Vanderbilt Institute for Clinical and Translational Research, Vanderbilt University, Nashville, USA) and stored securely to maintain data confidentiality and integrity.

Clinical Outcomes

(1) Based on a standard oral glucose tolerance test (OGTT) conducted between 24 and 28 weeks of gestation, the study's main outcome was the incidence of GDM, which was defined as the percentage of women diagnosed with GDM in the myo-inositol (intervention) group compared to the placebo (control) group; and (2) Secondary outcomes encompass both maternal and neonatal outcomes of clinical relevance in the care of pregnant women with diabetes in pregnancy. The maternal outcomes included fasting insulin level, gestational weight gain, polyhydramnios, rate of Caesarean section, Homeostatic Model Assessment of Insulin Resistance (HOMA IR) and Homeostatic Model Assessment of β-cell Function (HOMA B) levels. The neonatal outcomes were birth weight, neonatal hypoglycemia, respiratory distress, and admission to the NICU.

Insulin resistance and β-cell function were assessed using the HOMA-IR was calculated as: fasting insulin (µIU/mL) × fasting glucose (mmol/L) / 22.5. HOMA-B was calculated as: (20 × fasting insulin (µIU/mL)) / (fasting glucose (mmol/L) − 3.5).

Statistical analysis

Sample Size

The sample size was calculated to detect a 40% reduction in the incidence of GDM, accounting for a 20% dropout rate, with a final target of 640 participants, having 320 in the intervention arm and 320 in the control arm. Based on previously published randomized controlled studies assessing the impact of myo-inositol supplementation on the incidence of gestational diabetes mellitus, the sample size was determined. A total sample size of 640 people (320 each group) was needed to obtain 80% power with a two-sided alpha of 0.05, assuming a decrease in GDM incidence from 33% in the control group to 18% in the intervention group, as reported by D'Anna et al. An estimated attrition rate of about 10% was taken into consideration in this computation [6]. The study's early termination due to financial limitations led to a significantly smaller sample size than anticipated.

Statistical Methods

Descriptive and inferential statistics were applied, including frequencies, proportions, means, medians, standard deviations, and quartiles. T-tests, chi-squared tests, and logistic regression analyses were performed using R software (R Foundation for Statistical Computing, Vienna, Austria) and SAS v9.4 (SAS Institute Inc., Cary, North Carolina, USA) and a significance level of p<0.05 was used for all tests.

Forty-three participants, around 7% of the intended sample, completed the study as a result of the trial's early termination due to financial limitations, despite the sample size calculation being done beforehand. At the time of discontinuation, neither a formal futility analysis nor a conditional power analysis was carried out. As a result, only significant effect sizes would have been discernible with the attained sample size, and the study was significantly underpowered to identify slight between-group variations in the incidence of GDM. Therefore, rather than being confirming, the data should be seen as exploratory and hypothesis-generating.

## Results

Maternal characteristics

To compare the myo-inositol and placebo groups, baseline maternal characteristics were evaluated (Table 1).

**Table 1:** Characteristics of the two maternal study groups BMI, body mass index; GWG, gestational weight gain; DM, diabetes mellitus; BW, birth weight; GDM, gestational diabetes mellitus; PCOS, polycystic ovary syndrome; HOMA-B, homeostatic model assessment of β-cell function; HOMA-IR, homeostatic model assessment of insulin resistance; Hx, history; FHx, family history.

	Myo-inositol (n=18)	Placebo (n=25)	p-value
Age (years)	33.7 ± 4.7	33.5 ± 5.3	0.84
BMI (kg/m²)	27.9 ± 4.2	25.3 ± 4.5	0.06
Parity, mean (number of children, SD)	1.9 (1.5)	1.6 (1.9)	0.590
GWG, mean (kg/week, SD)	1.08 (0.85)	1.17 (0.97)	0.802
Gestational weight gain (kg)	11.4 ± 4.9	12.6 ± 7.22	0.61
Obesity (n, %)	5 (27.7%)	4 (16 %)	0.021
GWG/Week (kg/week)	0.30 ± 0.128	0.33 ± 0.183	0.62
Qatari (n, %)	7 (38.5%)	6 (24.0%)	0.688
Non-Qatari (n, %)	11 (61.5%)	19 (76.0%)	
FHx of DM (n, %)	10 (55.5%)	9 (36 %)	0.379
Hx of BW >4 kg (n, %)	1 (5.6%)	0 (0.0%)	0.448
Hx of GDM (n, %)	3 (16.6%)	2 (8%)	0.632
Hx of thyroid disorders (n, %)	0 (0.0%)	3 (12%)	0.232
Hx of PCOS (n, %)	0 (0.0%)	2 (8%)	0.488
Hx of miscarriage (n, %)	4 (22.2%)	6 (24%)	1.00
Fasting glucose (mmol/L)	5.0 ± 0.41	4.86 ± 0.38 (n=17)	0.129
1 hr glucose (mmol/L)	8.0 ± 2.78	7.68 ± 1.88	0.73
2 hr glucose (mmol/L)	6.98 ± 2.48	6.67 ± 1.89	0.73
Fasting Insulin (µIU/mL)	11.38 ± 7.11	8.65 ± 5.01	0.28
HOMA B (%)	134.48 ± 68.93	124.03 ± 57.88	0.69
HOMA IR (index)	2.66 ± 1.76	1.93 ± 1.28	0.24

Maternal age, mean BMI, gestational weight gain, parity, nationality, and other high-risk obstetric factors did not differ statistically significantly across the groups. However, compared to the placebo group, the myo-inositol group had a substantially greater prevalence of obesity (BMI >30 kg/m^2^), a major risk factor for GDM (27.7% vs. 16.0%, p=0.021). This suggests a baseline imbalance that may have affected metabolic results and how the treatment benefits were interpreted.

Primary outcome

Comparing the incidence of GDM between the myo-inositol and placebo groups was the study's main finding. Eighteen women in the myo-inositol group and 25 women in the placebo group had GDM, although there was no statistically significant difference between the groups. The incidence of GDM did not differ statistically significantly between the myo-inositol and placebo groups. The OGTT revealed no statistically significant differences between the groups in their fasting, one-hour, or two-hour plasma glucose levels. Furthermore, fasting insulin levels, HOMA-B, and HOMA-IR did not change significantly between the intervention and placebo groups.

Secondary outcome

The myo-inositol and placebo groups were evaluated for secondary maternal and neonatal outcomes (Table 2).

**Table 2:** Maternal and neonatal outcomes SD, standard deviation; NICU, Neonatal Intensive Care Unit.

	Myo-inositol (n=18)	Placebo (n=25)	p-value
Birth weight (kg)	3.15 (0.66)	3.08 (0.77)	0.225
Gestational age, mean (SD)	38.0 (2.9)	37.8 (3.1)	0.868
Induction of labor	3 (16.6%)	4 (16%)	1.00
C-section	7 (38.9%)	10 (40%)	
Assisted vaginal delivery	0 (0.0%)	1 (4%)	
Polyhydramnios	2 (11.1%)	0 (0.0%)	0.192
NICU admission	3 (16.7%)	3 (12%)	1.00
Neonatal hypoglycaemia	1 (5.5%)	0 (0.0%)	0.448
Respiratory distress	3 (16.7%)	3 (12.3%)	0.632

Birth weight, gestational age at delivery, induction of labor, method of delivery, polyhydramnios, admission to the NICU, newborn hypoglycemia, and respiratory distress did not differ statistically significantly across the groups.

Post-hoc exploratory analysis: GDM versus non-GDM groups

To identify the metabolic differences between women who acquired GDM (n=16) and those who did not (n=27), a post-hoc exploratory study was carried out (Table 3).

**Table 3:** Post-hoc exploratory analysis of the groups with or without GDM BMI, body mass index; GWG, gestational weight gain; HOMA-B, homeostatic model assessment of β-cell function; HOMA-IR, homeostatic model assessment of insulin resistance.

	GDM (n=16)	No GDM (n=27)	p-value
Age (years)	34.1 ± 5.3	33.6 ± 5.3	0.81
BMI (kg/m²)	27.8 ± 5.7	25.9 ± 4.2	0.29
Gestational weight gain (kg)	10.0 ± 6.3	13.1 ± 6.13	0.21
GWG/Week (kg)	0.27 ± 0.17	0.33 ± 0.15	0.38
Fasting glucose (mmol/L)	5.24 ± 0.35	4.67 ± 0.99	0.001
1 hr glucose (mmol/L)	9.18 ± 2.82	5.78 ± 1.00	0.0007
2 hr glucose (mmol/L)	8.45 ± 2.35	5.67 ± 0.99	0.0007
Fasting Insulin (µIU/mL)	12.65 ± 7.29	7.37 ± 3.24	0.027
HOMA B (%)	132.29 ± 71.49	125.09 ± 54.4	0.78
HOMA IR (index)	3.05 ± 1.82	1.54 ± 0.69	0.010
Gestational age (weeks)	37.8 ± 3.89	39.21 ± 0.57	0.03
Birth weight (kg)	3.27 ± 0.82	3.39 ± 0.53	0.8

Women with GDM showed increased insulin resistance as determined by HOMA-IR (3.05 ± 1.82 vs. 1.54 ± 0.69, p=0.010) and substantially higher fasting insulin levels (12.65 ± 7.29 µIU/mL vs. 7.37 ± 3.24 µIU/mL, p=0.027). There was no discernible variation in HOMA-B across the groups. Additionally, the GDM group gave birth at a somewhat earlier gestational age than the non-GDM group (37.8 ± 3.89 vs. 39.21 ± 0.57 weeks, p=0.03). This investigation was intended to characterize anticipated metabolic variations within the trial group rather than to evaluate the impact of myo-inositol.

The obstetric and neonatal outcomes for women with and without GDM are contrasted in Table 4.

**Table 4:** Obstetric and neonatal outcomes in GDM versus non-GDM GDM, Gestational diabetes mellitus; NICU, Neonatal Intensive Care Unit.

	GDM (n=16)	No GDM (n=27)	p-value
Induction of labor	4 (25 %)	5 (18.5%)	0.646
C-section	7 (43.8%)	12 (44.4%)	
Assisted vaginal delivery	0 (0.0%)	1 (3.7%)	
Polyhydramnios	2 (11.1%)	0 (0.0%)	0.135
NICU admission	4 (25%)	5(18.5%)	0.164
Neonatal hypoglycemia	1 (5.6%)	0 (0.0%)	0.379
Respiratory distress	3 (18.7%)	4 (14.8%)	0.339

Regarding induction of labor, method of delivery, polyhydramnios, NICU hospitalization, newborn hypoglycemia, and respiratory distress, no statistically significant differences were found between the two groups. The study was not powered to evaluate differences in obstetric or neonatal outcomes between these groups, thus these results, which are given as part of the post-hoc exploratory analysis, should be considered cautiously.

## Discussion

This randomized, double-blind, placebo-controlled pilot trial evaluated the effect of antenatal myo-inositol supplementation on the incidence of GDM and other maternal and neonatal outcomes in a Qatari population. Although the study was terminated before reaching the planned sample size due to funding constraints, analysis of the available data from 43 participants who completed the trial revealed no statistically significant differences between the myo-Inositol and placebo groups in the incidence of GDM or in secondary maternal and neonatal outcomes. However, funding limitations forced the early termination of this study, which left it noticeably underpowered. As a result, the current pilot study was unable to sufficiently test its initial hypothesis or confirm or rule out a clinically significant impact of myo-inositol supplementation on the incidence of GDM. Therefore, the lack of statistically significant changes can be considered as inconclusive, and no firm conclusions about myo-inositol's effectiveness in this population can be made.

Although differences in baseline risk profiles, study power, and intervention methods limit meaningful comparison, our results are generally consistent with other earlier trials that reported no discernible benefit of myo-inositol supplementation. Notably, baseline risk profiles, such as the incidence of obesity, family history of diabetes, ethnicity, and metabolic traits of recruited participants, have varied significantly among trials indicating a protective effect of myo-inositol. On the other hand, null results have been reported in a number of investigations that were carried out in diverse or lower-risk groups. These variations in baseline GDM risk, as well as variations in supplementation timing and dosage schedules, may help explain some of the contradictory findings between the studies and restrict direct comparison with benefit-reporting trials. Some studies found no reduction in GDM incidence among low-risk women receiving myo-inositol compared with placebo [13,14]. Importantly, our study focused on a high-risk population, yet similarly, did not demonstrate any effect on maternal weight gain, a finding that contrasts with or remains inconclusive when compared to results from some other studies [15,16].

Neonatal outcomes in our study, including birth weight, rates of macrosomia, and NICU admissions, were also comparable between groups, echoing the findings from a previous study [17]. Furthermore, no difference was observed in the rate of preterm birth, consistent with previous studies in the literature, such as the trial by D'Anna et al., which found no significant reduction in preterm delivery rates with myo-inositol supplementation [6,18].

The myo-inositol group's much greater baseline BMI is a major source of possible bias in this study. It may be more challenging to show that myo-inositol has a protective effect because obesity is a known risk factor for insulin resistance and GDM. As a result, the increased underlying metabolic risk in the intervention group may have reduced or obscured any possible effect of the supplementation. The baseline BMI imbalance, with a greater frequency of obesity in the myo-inositol group, is another drawback. This imbalance might have created bias and complicated treatment effects because obesity, insulin resistance, and GDM are strongly correlated. Crucially, rather than inflating advantage, this mismatch is likely to skew results in favor of the null.

We also observed no significant impact on maternal metabolic parameters, including insulin resistance and lipid profiles, which aligns with the findings of Santamaria et al. [16]. The prevalence of notable lifestyle behavior variations across the groups is a crucial factor to take into account when evaluating the trial's null results. Women in the myo-inositol group participated in better habits, such as greater physical activity, higher intake of legumes and beans, and reduced consumption of added sugars, according to secondary analysis of the Myo-Inositol for the Prevention of Gestational Diabetes Mellitus (MiGDM) study. A genuine impact of myo-inositol supplementation may be obscured, or findings may be skewed toward the null by these lifestyle variations, which constitute a significant confounding factor and may have independently improved glycemic outcomes. These results suggest that, at least in our study population, myo-inositol supplementation did not confer measurable metabolic or perinatal benefits.

Several factors may explain the lack of significant effects. First, our study was underpowered due to early termination and a smaller-than-planned sample size, limiting the ability to detect modest differences [13]. Second, variations in baseline risk for GDM, supplementation protocols (dose, timing, and duration), and study design across trials may contribute to inconsistent findings in the literature. Third, biological variability, including genetic, metabolic, and environmental influences, may affect individual responses to myo-inositol, potentially diluting any population-level effect [19]. Compared to the placebo group, women in the myo-inositol arm exhibited significantly healthier lifestyle behaviors, such as walking more (p=0.004), eating more legumes and beans (p=0.008), and consuming less added sugar (40.5% vs. 66.7%, p=0.014), according to a secondary analysis of the MiGDM trial. However, these improvements did not result in a lower incidence of GDM [20].

Strengths and limitations

The study employed a robust randomized controlled trial methodology, minimizing selection bias and enhancing internal validity. Double blinding of participants and investigators reduced the risk of performance and detection bias. This is among the few studies examining myo-inositol supplementation in a Middle Eastern population, contributing valuable regional data to the global evidence base.

Early termination due to funding constraints resulted in an underpowered study, limiting the ability to detect small but potentially clinically meaningful effects, and loss to follow-up reduced the number of participants completing the trial. In addition, findings may not be generalizable to other populations with different demographic or clinical characteristics. The study did not stratify participants by baseline GDM risk, which may have masked potential benefits in higher-risk subgroups.

This research has a number of significant drawbacks. First, the sample size was significantly decreased due to early termination, which significantly limited statistical power. The study was unlikely to find anything other than significant treatment effects because no formal futility or conditional power analysis was done at the time of discontinuation. Second, there was a notable baseline disparity in the prevalence of obesity between the groups, even after randomization. Because obesity is a significant independent risk factor for GDM, the myo-inositol arm's higher obesity incidence may have skewed the results in favor of the null hypothesis. Causal conclusion about the impact of myo-inositol supplementation was limited because statistical adjustment for obesity was not possible due to the small sample size.

One of the study's main limitations is the significant disparities in lifestyle patterns between the groups. The myo-inositol group's higher levels of physical activity and healthier eating habits may have complicated the link between supplementation and glycemic outcomes, making it more difficult to interpret the null results and perhaps hiding a real treatment effect. Finally, the results have limited generalizability to other settings with differing clinical or demographic features because it was a small pilot research carried out in a particular population.

## Conclusions

In this randomized, double-blind, placebo-controlled pilot trial, antenatal myo-inositol supplementation did not significantly alter the incidence of GDM or the outcomes for mothers and newborns when compared to a placebo. However, this study was unable to confirm or rule out a clinically significant impact because of early termination and severe underpowering. Hence, the results should be regarded as equivocal rather than proof of ineffectiveness. Higher fasting insulin levels, greater insulin resistance (HOMA-IR), and lower gestational age at delivery are among the observed differences between women who developed GDM and those who did not. These differences are expected metabolic associations that support the biological plausibility of the study hypothesis and validate that the study was appropriately designed to address the research question.

To conclusively ascertain whether myo-inositol supplementation has a role in preventing GDM and improving perinatal outcomes in this and similar populations, larger, sufficiently powered, multicenter randomized trials with stratification by baseline GDM risk and strict control of lifestyle confounding are necessary due to the high likelihood of a type II error.
